# Effects of rice blast biocontrol strain *Pseudomonas alcaliphila* Ej2 on the endophytic microbiome and proteome of rice under salt stress

**DOI:** 10.3389/fmicb.2023.1129614

**Published:** 2023-03-07

**Authors:** Qingchao Zeng, Xiaowu Man, Zeyang Huang, Lubo Zhuang, Hanmeng Yang, Yuexia Sha

**Affiliations:** ^1^Institute of Plant Protection, Ningxia Academy of Agriculture and Forestry Sciences, Yinchuan, China; ^2^Beijing Advanced Innovation Center for Tree Breeding by Molecular Design, Beijing Forestry University, Beijing, China; ^3^Institute of Plant Protection, Beijing Academy of Agricultural and Forestry Sciences, Beijing, China; ^4^Department of Plant Pathology, MOA Key Lab of Pest Monitoring and Green Management, College of Plant Protection, China Agricultural University, Beijing, China

**Keywords:** rice, salt stress, *Pseudomonas*, endophytic, microbiome, proteomic

## Abstract

**Introduction:**

Soil salinity is a prevalent environmental stress in agricultural production. Microbial inoculants could effectively help plants to alleviate salt stress. However, there is little knowledge of the biocontrol strain *Pseudomonas alcaliphila* Ej2 mechanisms aiding rice plants to reduce the adverse effects caused by salt stress.

**Methods:**

We performed integrated field and greenhouse experiments, microbial community profiling, and rice proteomic analysis to systematically investigate the Ej2 mechanism of action.

**Results:**

The results displayed that biocontrol strain Ej2 increased shoot/root length and fresh/dry weight compared with control under salt stress. Meanwhile, strain Ej2 has the ability to control rice blast disease and promote rice growth. Furthermore, the microbial community analysis revealed that the alpha-diversity of Ej2-inoculated plants was higher than the control plants, expect the Shannon index of the bacterial microbiome and the Ej2-inoculated samples clustered and separated from the control samples based on beta-diversity analysis. Importantly, the enriched and specific OTUs after Ej2 inoculation at the genus level were *Streptomyces*, *Pseudomonas*, *Flavobacterium*, and *Bacillus*. Moreover, we observed that Ej2 inoculation influenced the rice proteomic profile, including metabolism, plant-pathogen interactions, and biosynthesis of unsaturated fatty acids. These results provide comprehensive evidence that Ej2 inoculation induced the rice endophytic microbiome and proteomic profiles to promote plant growth under salt stress.

**Discussion:**

Understanding the biocontrol strain effects on the endophytic microbiome and rice proteomics will help us better understand the complex interactions between plants and microorganisms under salt stress. Furthermore, unraveling the mechanisms underlying salt tolerance will help us more efficiently ameliorate saline soils.

## Introduction

Climate change, a very important ongoing topic, has affected our planet in different ways. Crop productivity is affected by climate change, with serious implications for food security (Vaishnav et al., 2019; Nawaz et al., 2020). Among them, soil salinity is one of the current major and widespread agricultural challenges that hinder global food security (Mukhopadhyay et al., 2021). An almost 70% yield loss has been reported in cereal crops, such as rice, wheat, and maize, due to soil salinity and sodicity (Rajendran et al., 2009; Nawaz et al., 2020). Moreover, 20% of total cultivated and 33% of irrigated agricultural lands worldwide are afflicted by soil high salinity (Shrivastava and Kumar, 2015). Several strategies, including plant genetic engineering and the utilization of plant growth-promoting bacteria, have been investigated and implemented to decrease the adverse effects caused by high salinity on plant growth (Vaishnav et al., 2019). Although effective, plant genetic engineering is time-consuming and cost-intensive. The application of beneficial microorganisms represented a useful approach to increase salt tolerance (Berg, 2009; Vaishnav et al., 2019). Most bacterial genera, including *Agrobacterium*, *Bacillus*, *Pseudomonas*, and *Rhizobium*, have frequently been reported to improve plant salt tolerance. *Pseudomonas syringae* and *Pseudomonas fluorescens* increased plant growth, yield, and leaf chlorophyll content under salt stress (Vimal et al., 2018; Nawaz et al., 2020). Meanwhile, *Bacillus* could promote proline biosynthesis in pepper plants under osmotic stress (Sziderics et al., 2007). To improve the tolerance and increase the yield of crop exposed to salinity stress, it is necessary to determine the underlying acclimatization and tolerance mechanisms.

Plants hosts a plethora of microorganisms, the plant microbiota (Vogel et al., 2022). There is increasing evidence indicating that the microbiome impacts host performance, such as plant growth promotion, nutrient uptake, increased abiotic stress tolerance, and pathogen growth inhibition (Cadot et al., 2021; Schmitz et al., 2022). More evidence indicates that the plant microbiome is shifting upon biotic and abiotic influences such as pathogen infection and salt and drought stresses. Importantly, wheat plants infected by pathogens could recruit beneficial microbes to protect the plants and maintain plant growth (Kusstatscher et al., 2019; Yuan et al., 2019; Liu et al., 2021a; Li et al., 2022). Meanwhile, the key plant growth-promoting bacteria of garlic such as *Pseudomonas* could promote plant growth (Zhuang et al., 2021). Furthermore, *Sphingomonas* and *Microcoleus* as the predominant genera in salt-treated rhizosphere soils as plant growth-promoting rhizobacteria might enhance salt tolerance (Xu et al., 2020). Moreover, organic fertilizers altered the plant microbiome and promote the enrichment of beneficial microorganisms. For example, bacteriophages could control pathogens and they do not alter the existing rhizosphere microbiome and enrich bacterial species to inhibit pathogen growth and virulence (Wang X. et al., 2019). Meanwhile, bio-organic fertilizers promote *Ralstonia solanacearum* suppression, inducing changes in community composition rather than only through the abundance of the introduced strains (Deng et al., 2022). Microorganisms play a vital role in protecting plants against multiple environmental stresses. Importantly, bacterial inoculation has limited success in the field, partially due to the inoculants being outcompeted by the native microbial communities in plants (French et al., 2021). Although the mechanism of action of individual microorganisms has been investigated thoroughly, their impact on plant endophytic microbiome composition is still not fully explored.

Plants can also cope with different stress conditions. Plants have a highly coordinated immune system in which different physiological mechanisms are involved in signaling cascades and induce stress tolerance (Vaishnav et al., 2019). Salt stress tolerance mechanisms are highly complex, and pathways are coordinately linked (Culligan et al., 2012). Importantly, plant-associated microbes have key roles contributing to plant survival under stress conditions. The beneficial effects evoked by endophytes are osmotic adjustment, detoxification, phytohormone regulation, and nutrient acquisition, alleviating salt stress negative effects (Vaishnav et al., 2019). For example, bacterial ACC deaminase activity reduced the host plants’ salinity-induced oxidative and osmotic damage. Specifically, *P. migulae* 8R6 can produce ACC deaminase and ameliorate salt stress in tomato (Ali et al., 2014). Meanwhile, *P. pseudoalcaligenes* inoculation induces glycine betaine accumulation, which improves salinity tolerance in rice plants (Jha et al., 2011). At the same time, the microbes induce plant stress resistance by enhancing antioxidant enzymes activity and other non-enzymatic antioxidants (Gururani et al., 2013). Salt and bacterial inoculation in canola increased photosynthesis, antioxidative processes, transportation across membranes, and pathogenesis-related proteins, according to a proteome analysis (Cheng et al., 2012). Furthermore, the bacterial associations increased nutrients (N, P, K, Ca, and Mg) and decreased sodium accumulation under salinity. Stress-responsive genes include dehydration-responsive element binding protein, ethylene-responsive factor, high-affinity K^+^ transporter, and pyrroline-5-carboxylase synthetase (Vaishnav et al., 2019). During salt stress, plants accumulate stress-responsive proteins to combat the adverse effects of osmotic and ionic stress. However, there is little knowledges of how the *Pseudomonas alcaliphila* strain Ej2 help rice plants alleviate salt stress.

Rice is a model crop used in research for saline-alkali land improvement. High soil salinity reduces plant yield and thus causes major economic losses. It is important to understand rice responses to salt stress comprehensively. In this study, we isolated the endophytic *P. alcaliphila* Ej2 from rice leaves. This strain inhibited *Magnaporthe oryzae* growth and promoted rice growth under salt stress. This objective of this study was to investigate the *P. alcaliphila* mechanism of action on rice growth and yield enhancement under salt stress. To address these questions, we used metabarcoding sequencing and proteomic analysis to determine how strain Ej2 affects the endophytic microbiome and protein expression in rice under salt stress. We aimed to address (1) the endophytic microbiome taxonomic differences between inoculated and control plants; (2) how Ej2 inoculation affects the protein expression in rice roots under salt stress. Understanding the effects of the Ej2 biocontrol strain on rice endophytic microbiome and proteome will help us to better understand the complex interactions between plants and microorganisms under salt stress. Furthermore, elucidating the mechanisms underlying salt tolerance in plants will aid the more efficient saline soil amelioration.

## Materials and methods

### Strains and growth conditions

The strain *Pseudomonas alcaliphila* Ej2 and pathogens *Fusarium solani* N18-1-2, *F. moniliforme* N19-2-2, *F. oxysporum* f.sp. *niveum* M8, Colletotrichum gloeosporioides ZDP21, Alternaria alternata f.sp. mali BJ-A5, *A. alternata* BJ-ST24, and *M. oryzae* P131 were used in this study. The biocontrol strain Ej2 was isolated from rice leaf (Ninggeng No. 48) which collected from Pingluo, Ningxia (38.26–38.91°N, 105.53–106.52°E) (Sha et al., 2021). All the pathogen were donated by China Agricultural University and Institute of Plant Protection, Beijing Academy of Agricultural and Forestry Sciences. They were stored at the Institute of Plant Protection, Ningxia Academy of Agriculture and Forestry Sciences, Yinchuan, China. The bacterial and fungal strains were cultured in King’s B (KB, Proteose peptone No.3, 10 g/L; K_2_HPO_4_, 1.5 g/L; MgSO_4_·7H_2_O, 1.5 g/L; glucose, 20 g/L; and agar, 18 g/L) and potato dextrose agar (PDA, potato 200 g/L; glucose 20 g/L; and agar, 18 g/L) plate, respectively. Then, the fungal strains incubated at 30°C for 5–7 days and the bacterial strain grown at 28°C for 24 h (Zhao et al., 2019; Liu et al., 2021b).

### Strain identification

Bacterial strains Ej2 were identified based on their 16S rRNA gene sequences. Firstly, the overnight cultures of Ej2 were harvested in a microcentrifuge tube at 13,000 × g for 2 min. Then, the total DNA of Ej2 was extracted using DNA extraction kit based on the instruction (Accurate, Hunan, China). The 16S rDNA sequence was amplified using the universal primer 27F (5′-AGAGTTTGATCCTGGCTCAG-3′) and 1492R (5′-GGTTACCTTGTTACGACTT-3′) primers (Shanghai Sangon Biotech Co., Ltd) (Zhao et al., 2019). All PCR reactions were performed in 5 μl of 10× Taq buffer, 4 μL of 2.5 mM dNTPs, 1 μL of each primer (10 μM), 2 μL of bacteria DNA, 0.5 μL of Taq DNA polymerase, and ddH2O to a 50 μL final volume. Thermo cycler conditions were 5 min at 95°C; 30 cycles of 95°C for 30 s, 55°C for 30 s, and 72°C for 90 s; 72°C for 10 min. The PCR products were sequenced by Majorbio Co. Ltd., Shanghai, China. BLAST was performed with the resulting sequences against the National Center for Biotechnology Information (NCBI) database. Then, the homologous sequences were downloaded from NCBI database. Next, the highly homologous sequences were selected for multiple sequence alignment using CLUSTAL X in the MEGA 7.0 software (Kumar et al., 2016). A phylogenetic tree was constructed *via* a Neighbor-Joining (NJ) approach, with 1,000 replicate Bootstrap analyzes used to calculate node support (Chen et al., 2016).

### Antifungal assays

The dual culture technique was used to detect Ej2 antagonistic activities toward pathogenic fungi. The pathogens were cultured on PDA medium for 5–7 days, and then a 10 mm mycelial plug was placed in the center of a fresh PDA plate (Liu et al., 2021b). The tested bacteria were streaked around the fungal plug and cultured at 28°C for 5 days to examine potential antagonistic effects. Three replicates were performed for the assay. Plates that were only inoculated with pathogenic fungi served as negative controls. The antagonistic effect was assessed by measuring the inhibition zones and colony diameters (Liu et al., 2021b).

### Evaluation of biocontrol efficacy of Ej2 under greenhouse and field condition

The Ej2 strain biocontrol efficacy was tested using spray inoculation on rice cultivar G19 (Sha et al., 2020). Rice seeds were surface sterilized with 1% sodium hypochlorite solution for 30 min and were subsequently washed with autoclaved distilled water five times. For inoculum preparation, a single Ej2 colony grown on KB medium in plates was transferred to 5 ml KB broth and incubated at 30°C on a shaker (200 rpm) for 24 h. The grown culture was harvested by centrifuging at 5000 rpm for 5 min at 4°C, and the cell pellets were resuspended in phosphate-buffered saline (PBS) to obtain an optical density of ~1.0 at 600 nm (~1.0 × 10^8^ CFU/mL). For the greenhouse experiments, after 20 days of the plantation, the rice plants were inoculated with 50 ml (1 × 10^8^ CFU/ml) by leaf-spraying, tricyclazole (75% WP, 1:1000 dilution), and sterile water used as a positive and negative control. Then, 20 ml of *M. oryzae* suspension (1 × 10^6^ conidia/mL) was spray inoculated on the rice plants after 24 h. The experiment was performed in 4 replicates per treatment, with 90 seedlings per replicate. The disease incidence and severity were investigated at 7 days after inoculation.

The effects of the bacterial strain Ej2 on rice growth under salt stress were investigated in a controlled environment using rice cultivar Ningjing No.61. Field soil was brought to the greenhouse, mixed with nutrient soil (3:1 ratio), and used as the planting soil. The field soil physicochemical characteristics were: pH = 8.5; Phosphorus 5.5 mg/kg; Organic matter 5.7 g/kg; total nitrogen 0.5 g/kg. The bacterial Ej2 strain inoculation was performed by soaking the sterilized rice seeds in a bacterial suspension (1.0 × 10^8^ CFU/mL) for 6 h, while for the control treatment, seeds were soaked in autoclaved distilled water. Meanwhile, the seeds were dried under a clean bench. Then, the seeds soaked in 5 mL, 0.2% NaCI concentration, were germinated in Petri dishes for 48 h at 28°C. Subsequently, six seeds were sown in each plastic pot filled with soil. 25 days after sowing, the plants were uprooted. All samples were kept in cold conditions, transferred to the lab, and stored at −80°C before DNA extraction.

The field experiments were performed during the 2020 growing season in the main rice production fields in Lingwu (106.18 E, 37.59 N) and Wuzhong (106.14 E, 37.94 N) in Ningxia province, northwest China. The two sites are in a temperate continental climate zone. The average altitude is 1,133 m. The rice cultivars used were the same as those planted by local farmers, Qiuyou 88 and Jinggu No. 8. The field was separated into four plots, and each plot had an area of 600 m^2^. The Ej2 strain (>1 × 10^9^ CFU/mL, 7,500 mL added in 2400 L water for every hectare), tricyclazole (75% WP, 1200 g added in 750 L water for every hectare), and water were applied in the tillering, booting, and rupturing stage. The leaf and stem disease index of rice blast were calculated at tillering and maturity stage. The rice yield was investigated at the maturity stage. The experiment was performed with 4 replicates per treatment. The rice blast incidence and severity were quantified at the stem or leaf using a 0–9 scale, as reported previously (Sha et al., 2020). The disease index (DI) was calculated according to the following formulae: Disease index = [∑(Rating×the number of diseased leaves rated)/The total number of leaves × Highest rating] × 100. Disease incidence (%) = (Total number of diseased leaves/total number of investigated leaves) × 100. Moreover, the plant height, panicle weight, and thousand kernel weight were measured at the maturity stage for control and inoculation plants.

### DNA extraction and amplicon sequencing

The rice roots were thoroughly washed under distilled water. Then, the root samples were rinsed with 75% ethanol for 3 min. This procedure was repeated three times, and then the roots were finally washed five times with sterile water. The last wash water was inoculated on a nutrient agar plate and incubated for 48 h at 37°C. No viable colonies formed, which indicated that the disinfection procedure was efficient (Liu et al., 2021a). The roots were placed in liquid nitrogen and ground to a fine powder for DNA extraction. DNA was extracted using the Fast DNA™ SPIN Kit (MP Biomedicals) according to the manufacturer’s instructions. DNA concentration and quality were determined using a NanoDrop 2000 spectrophotometer (Thermo Scientific, United States).

The universal primers 799F/1193R and ITS1F/ITS2R were used to amplify the 16S and ITS genes to analyze the bacterial and fungal community (Gardes and Bruns, 1993; Caporaso et al., 2011). The PCR reaction was performed in 20 μL volume, containing 0.8 μL of both primers (5 M), 10 ng of template DNA, 4 μL of 5× TransStart FastPfu Buffer and 0.4 μl TransStart FastPfu DNA polymerase (TransGen, Beijing). Amplification was carried out with the following cycling conditions: 3 min initial denaturation at 95°C, followed by 27 cycles of 95°C for 30 s, 55°C for 30 s, and 72°C for 45 s, with a final 10 min elongation at 72°C. The PCR products were quantified using QuantiFluor™-ST (Promega, United States) and then diluted to reach an equal concentration. The library quality was assessed on the Qubit 2.0 Fluorometer (Thermo Scientific) and Agilent Bioanalyzer 2,100 system. The libraries were indexed using the TruSeq DNA Sample Prep Kits for Illumina. They were sequenced on an Illumina MiSeq platform at Majorbio Co., Ltd. (Shanghai, Beijing), and 250 paired-end reads were generated.

### Protein extraction, digestion, and TMT labeling

The rice root samples were ground into a fine powder in liquid nitrogen. Subsequently, the powder was suspended in a lysis buffer (1% sodium deoxycholate, 8 M urea) containing an appropriate protease inhibitor. The mixture was allowed to settle at 4°C for 30 min, during which the sample was vortexed every 5 min and was subsequently treated by ultrasound at 40 kHz and 40 W for 2 min. After centrifugation at 16,000× *g* at 4°C for 30 min, the protein supernatant concentration was determined by the bicinchoninic acid (BCA) method using BCA protein Assay Kit (Pierce, Thermo, United States). Protein quantification was performed according to the kit protocol.

100 μg of protein sample was taken, and the volume was replenished to 90 μl adding the lysate. Tris-(2-carboxyethyl)-phosphine (TCEP) (10 mM) was added, and the mixture was incubated at 37°C for 1 h. Then, iodoacetamide (40 mM) was added, and the mixture was incubated at room temperature in the dark for 40 min. Prechilled acetone (*v*:*v* = 6:1) was subsequently added and incubated for 4 h at-20°C before centrifugation at 10,000 g for 20 min. The pellet was re-suspended with 100 μl riethylammonium bicarbonate (TEAB, 50 mM) buffer. All collected proteins were transferred to a new tube, and trypsin digestion with a substrate ration of 1:50 (w/w) was performed at 37°C overnight, according to the manufacturer’s instructions.

The digested peptides were labeled with a TMT reagent kit according to the manufacturer’s instructions for TMT labeling (Thermo Fisher, United States). After tagging, the TMT-labeled samples were pooled and analyzed with a Thermo Scientific Vanquish F UHPLC system connected to a Q-Exactive hybrid quadrupole Orbitrap mass spectrometer (Thermo Fisher, United States). The TMT-tagged peptides were mixed and fractionated into fractions by ACQUITY Ultra Performance liquid chromatography (Waters, United States) with ACQUITY UPLC BEH C18 column (1.7 μm, 2.1 mm × 150 mm, Waters, United States) to increase proteomic depth. Briefly, peptides were separated first with an elution gradient (phase B: 5 mM ammonium hydroxide solution containing 80% acetonitrile, pH = 10) over 48 min at a flowrate of 200 μL/min. 20 fractions were collected from each sample, and were subsequently pooled, resulting in 10 total fractions per sample.

### LC–MS/MS analysis

Labeled peptides were analyzed by nano-flow liquid chromatography tandem mass spectrometry performed on a 9RKFSG2_NCS-3500R system (Thermo Fisher, United States) connected to a Q Exactive Plus Quadrupole Orbitrap mass spectrometer (Thermo Fisher, United States) through a nanoelectrospray ion source. Briefly, the C18 reversed-phase column (75 μm × 25 cm, Thermo Fisher, United States) was equilibrated with solvent A (A: 2% formic acid with 0.1% formic acid) and solvent B (B: 80% ACN with 0.1% formic acid). The peptides were eluted using the following gradient: 0–4 min, 0–5% B; 4–66 min, 5–23% B; 66–80 min, 23–29% B; 80–89 min, 29–38% B; 89–91 min, 38–48% B; 91–92 min, 48–100% B; 92–105 min, 100% B; 105–106 min, 100–0% B) at a flow rate of 300 nL/min. The Q Exactive Plus was operated in the data-dependent acquisition mode (DDA) to automatically switch between full scan MS and MS/MS acquisition. The full scan MS spectra (m/z 350–1,300) were acquired in the Orbitrap with 70,000 resolution. The automatic gain control (AGC) target was set 3e6, and the maximum fill time was 20 ms. Then, the top 20 precursor ions with the highest intensity were selected into collision cells for fragmentation by higher-energy collision dissociation (HCD). The MS/MS resolution was set at 35000 (at 100 m/z), the automatic gain control (AGC) target at 1e5, the maximum fill time at 50 ms, and dynamic exclusion was 18 s.

### Statistical analysis

Raw sequences were split according to their unique barcodes and trimmed of the adaptors and primer sequences using a proprietary script from the sequencing provider. Firstly, the paired-end reads were combined by the FLASH software (version 1.2.11) to obtain full-length sequences with default parameters (Magoc and Salzberg, 2011). The resulting sequences were processed using VSEARCH (version 11.0.667) and QIIME (version 1.9.1) (Caporaso et al., 2010; Zgadzaj et al., 2016). Quality control (fastq_maxee_rate = 0.01), singleton and chimera removal were performed for the resulting sequences. The sequence reads were then clustered into operational taxonomic units (OTUs) at a 97% similarity level using the UPARSE pipeline. Representative sequences were classified using the BLAST algorithm with SILVA v.13.2 and UNITE v8.0 reference databases (Quast et al., 2012; Nilsson et al., 2019). Mitochondrial and chloroplast DNA sequences and OTUs with a total relative abundance of <0.00001 in all samples were discarded. For the fungal community, only OTUs annotated at kingdom level were retained for analysis.

The significant differences were assessed with one-way ANOVA in GraphPad Prism 8. The OTU table was rarefied to reads (the lowest number was 25,273 and 62,294 for the bacterial and fungal community, respectively) for alpha-diversity and was normalized using the cumulative sum scaling (CSS) method for beta-diversity analysis. Both alpha-diversity and beta-diversity were calculated with the QIIME software. Permutational multivariate ANOVA (PERMANOVA) statistical tests were performed using the R package, vegan, with the adonis function having 1,000 permutations (Xiong et al., 2021). The Venn diagram was analyzed using the OECloud tools^1^ to analyze common and specific OTUs for control and treated samples. Using the STAMP software, Welch’s test calculated the significance of differences between control and treated samples (Parks et al., 2014). Differential abundance analysis was performed using EdgeR’s generalized linear model (GLM) approach. The differential OTUs with false discovery rate-corrected *p*-values <0.05 were identified as indicator OTUs (Robinson et al., 2010).

The RAW data files were analyzed using Proteome Discoverer (Thermo Scientific, Version 2.2) (Against the Mus_musculus database,^2^ Assembly Version GRCm38, 67,856 s). The MS/MS search criteria were as follows: Mass tolerance of 10 ppm for MS and 0.02 Da for MS/MS tolerance, trypsin as the enzyme allowing up to two internal trypsin cleavage sites missed, cysteine carbamidomethylation, the TMT of N-terminus and lysine side chains of peptides as fixed modifications, and methionine oxidation as dynamic modifications, respectively. The peptide identification false discovery rate (FDR) was set at FDR ≤ 0.01. A minimum of one unique peptide identification was used to support protein identification.

A total of 6,353 proteins expressed were identified in the rice root proteome in this study. The fold change (>1.2 or < 0.83) and *value of p* < 0.05 thresholds were used to identify differentially expressed proteins (DEPs). All identified proteins were annotated using GO^3^^,^^4^ and the KEGG pathway^5^. Then, DEPs were further used for GO and KEGG enrichment analysis. The proteomics data were analyzed on the online Majorbio Cloud Platform^6^.

## Results

### The *Pseudomonas alcaliphila* Ej2 strain is a potential biocontrol agent for *Magnaporthe oryzae*

**Figure 1:**
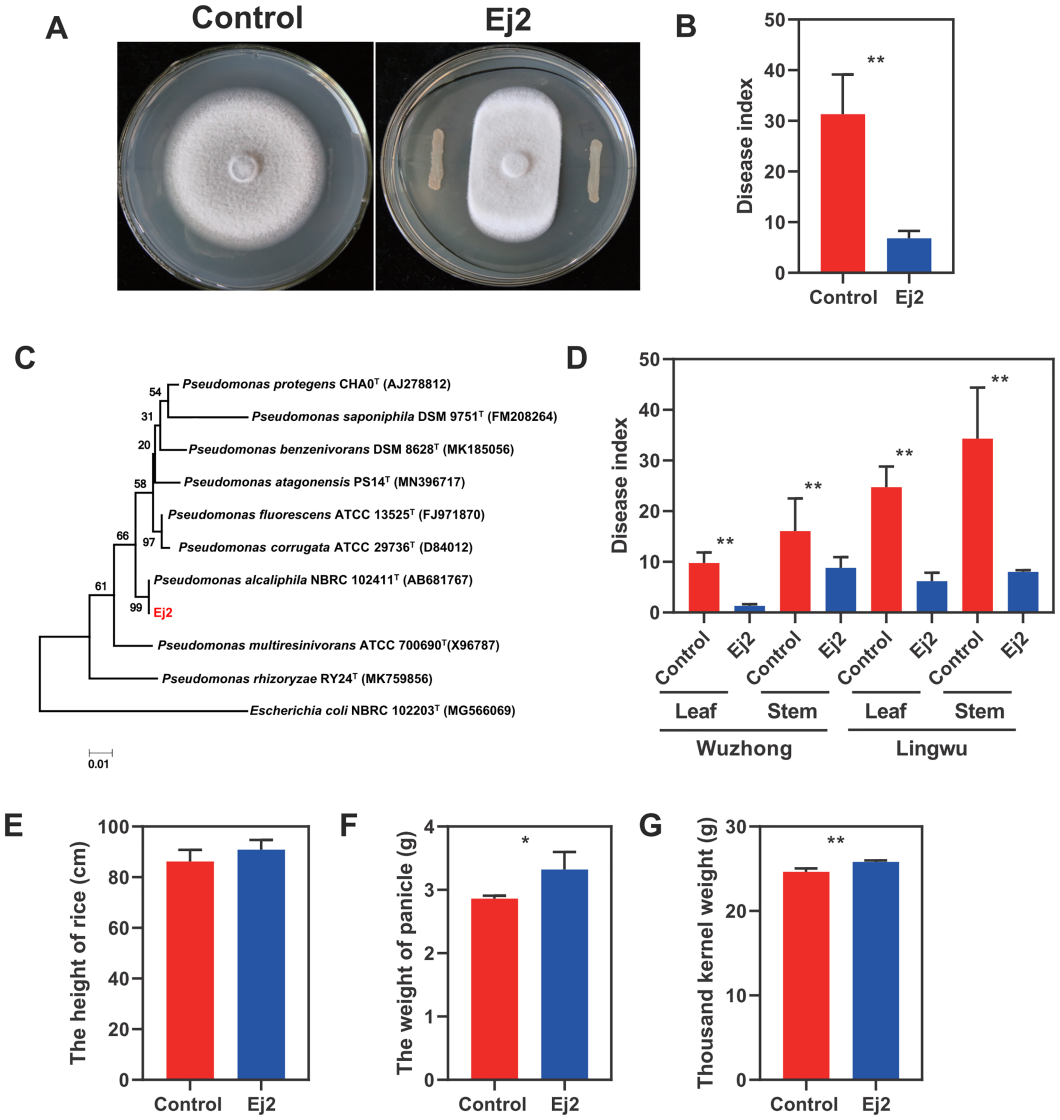
The general characteristics and growth-promoting traits of strain Ej2. **(A)** Inhibition of hyphal growth of *Magnaporthe oryzae* by strain Ej2. Left: *M. oryzae* CK. Right: *M. oryzae* at the presence of strain Ej2. The photograph was taken 5 days after inoculation. **(B)** Effect of strain Ej2 on rice blast disease index in the greenhouse experiment. **(C)** Phylogenetic tree based on 16S rRNA sequences obtained by the NJ method with 1,000 replicates. **(D)** Effect of strain Ej2 on rice leaf and stem blast disease index in a field trial. Rice yield attributes of Ej2-inoculated and control rice plants for **(E)** plant height, **(F)** weight panicle, and **(G)** thousand kernel weight. Each value represents the mean of four replicates ± standard error (SE). The significance test was performed based on a t-test using Prism 9. *p < 0.05, **p < 0.01.

The strain Ej2 was isolated from rice leaves and displayed obvious inhibition to *Magnaporthe oryzae* on petri dish assays (Figure 1A). Meantime, the results showed that Ej2 exhibited broad-spectrum antagonistic activities (Supplementary Table S1). Furthermore, the potential biological control abilities of Ej2 on rice blast disease were assessed in greenhouse experiments and field trials. We observed that Ej2 significantly decreased rice blast severity in pot experiments than leaves treated with water (Figure 1B, *p* < 0.01). In field trials, the rice leaf and stem blast disease indices in Ej2-treated plants were significantly reduced at Lingwu and Wuzhong sites compared to the control (*p* < 0.01, Figure 1D). In addition, we observed that the biocontrol efficiency in the Wuzhong field trial was higher compared to Lingwu. Thus, strain Ej2 showed excellent biocontrol efficiency in the field. Moreover, the growth-promoting traits of Ej2 were also examined. Rice yield attributes (the weight of panicle and thousand kernel weight) were significantly affected by Ej2 inoculation. However, the effect on plant height was not significant (Figures 1E–G). The above results showed that the strain Ej2 was a potential biocontrol candidate for the suppression of *M. oryzae*. Based on 16S rDNA sequence alignments, Ej2 exhibited a high similarity with *P. alcaliphila*. A phylogenetic tree was constructed based on closely related 16S rDNA sequences. The results showed that Ej2 was most closely related to *P. alcaliphila* (Figure 1C). Therefore, strain Ej2 was identified as *P. alcaliphila* (16S rDNA accession number: MN756644.1).

### Effects of strain Ej2 inoculation on the growth of rice plants under salt stress conditions

**Figure 2:**
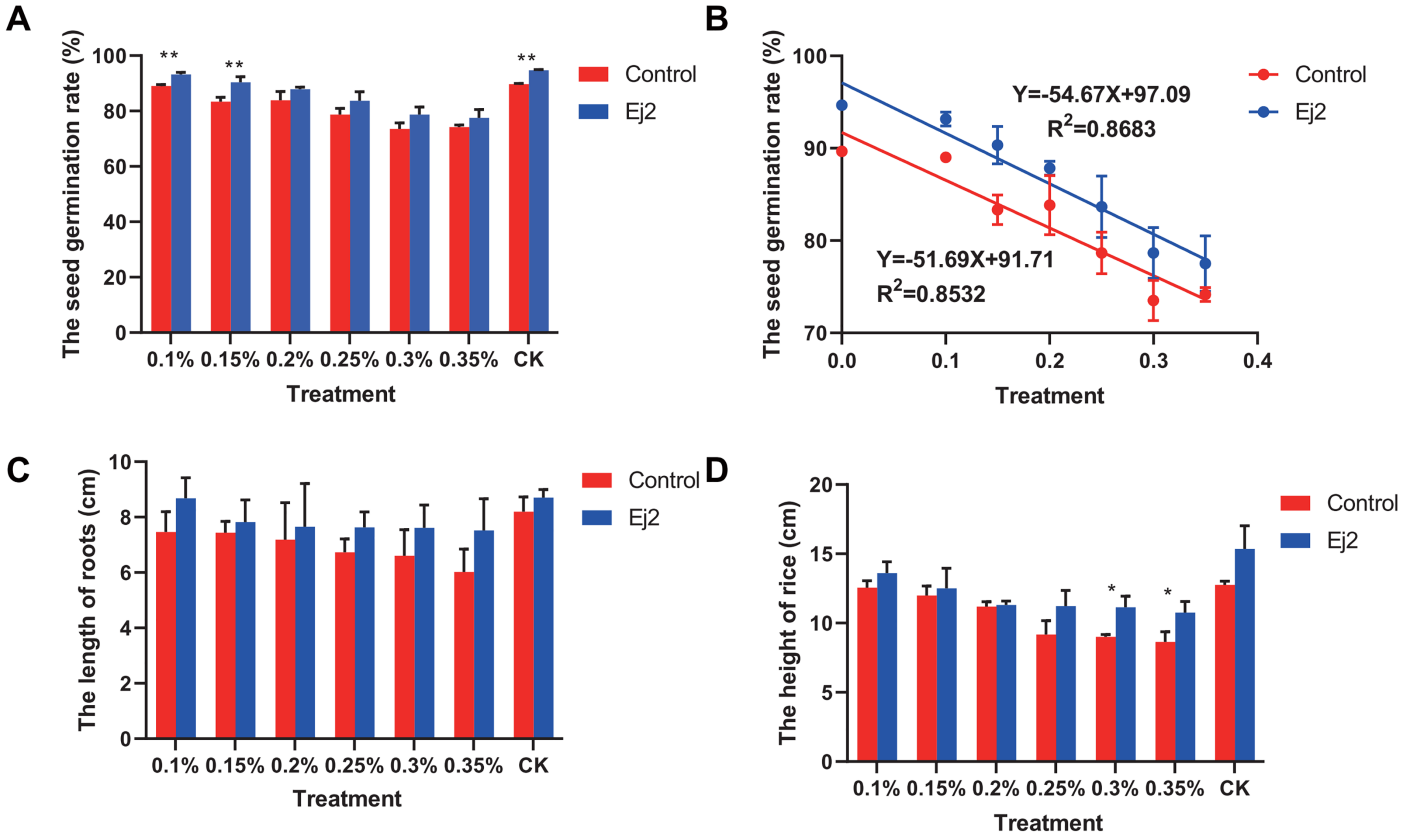
The rice growth-promoting capacity of strain Ej2 under different NaCI concentrations. **(A)** Rice seed germination after Ej2 inoculation and the control under different NaCI concentrations. **(B)** Correlation analysis between seed germination and different NaCI concentration in Ej2-inoculated and control plants. Effect of strain Ej2 on plant growth attributes **(C)** root length and **(D)** shoot height. Each value represents the mean of four replicates ± standard error (SE). The significance test was performed based on a t-test using Prism 9. *p < 0.05, **p < 0.01.

Different plant growth characteristics, mainly the seed germination, emergence rate, root length, shoot height, and root fresh and dry weight, were recorded compared to the control. In terms of seed germination, the results showed that the germination rate was improved compared to the control under different NaCl concentrations. Only the germination at NaCl concentration below 0.2% was significantly increased between Ej2-inoculated and control rice plants (Figure 2A). The seed germination responses were fitted and could be calculated by the regression equation *Y* = −54.67X + 97.09 (*R^2^* = 86.83) and *Y* = −51.69X + 91.71 (*R^2^* = 85.32%) for Ej2 inoculation and control (Figure 2B). The progressive NaCl concentration increase leads to a reduction in seed germination. Similar results were obtained for the emergence rate (Supplementary Figure S1A).

Strain Ej2 inoculation improved root length and shoot height at all levels of salt stress compared to the control. The Ej2 inoculation increased root length as compared to the control by 6.22%, 16.45, 5.13%, 6.51%, 13.30%, 15.33%, and 24.82% at 0, 0.1, 0.15, 0.2, 0.25, 0.3, and 0.35% NaCl concentration, respectively (Figure 2C). However, no significant differences in root length between Ej2 inoculation and control under salt stress were observed. Similarly, shoot height followed a similar trend as root length. The Ej2-inoculated plants exhibited an improvement in shoot height by 20.40%, 8.35%, 4.27%, 1.03%, 22.24%, 23.55%, and 24.43%, compared to control plants after 20 days later (Figure 2D). Notably, in control plants, the root fresh weight decreased with increasing NaCl concentrations, while in Ej2-inoculated plants, there was a noticeable enhancement of root fresh weight by 0.55, 6.61, 5.16, 4.33, 2.07, 2.85, and 5.07%, respectively, compared to control plants. The root dry weight was increased by 23.87% in Ej2-inoculated plants under 0.1% NaCl concentration compared to the control plants (Supplementary Figures S1B,C). Furthermore, leaf water content was improved in the Ej2-inoculated plants compared to control plants under different NaCl concentrations. The maximum differences were observed in Ej2-inoculated plants treated with 0.15% NaCl (Supplementary Figure S1D). These results indicated that strain Ej2 enabled the inoculated rice plants to grow better under salt stress compared to the control rice plants. Thus, strain Ej2 is a potent bioinoculants candidate to promote plant growth under saline conditions.

### Microbiome structure alterations between control and Ej2 inoculation rice plants

Bacterial and fungal microbiomes associated with rice roots were characterized by metabarcoding rDNA sequencing. Paired-end sequencing, after quality control, resulted in 667,275 and 844,120 high-quality reads. Sequences from each sample ranged from 50,510 to 153,032 and 70,231 to 172,381, with an average of 111,212 and 140,686 for the bacterial and fungal microbiome, respectively. Based on 97% similarity, these sequences were clustered into 1,533 and 158 OTUs for bacterial and fungal microbiomes.

**Figure 3:**
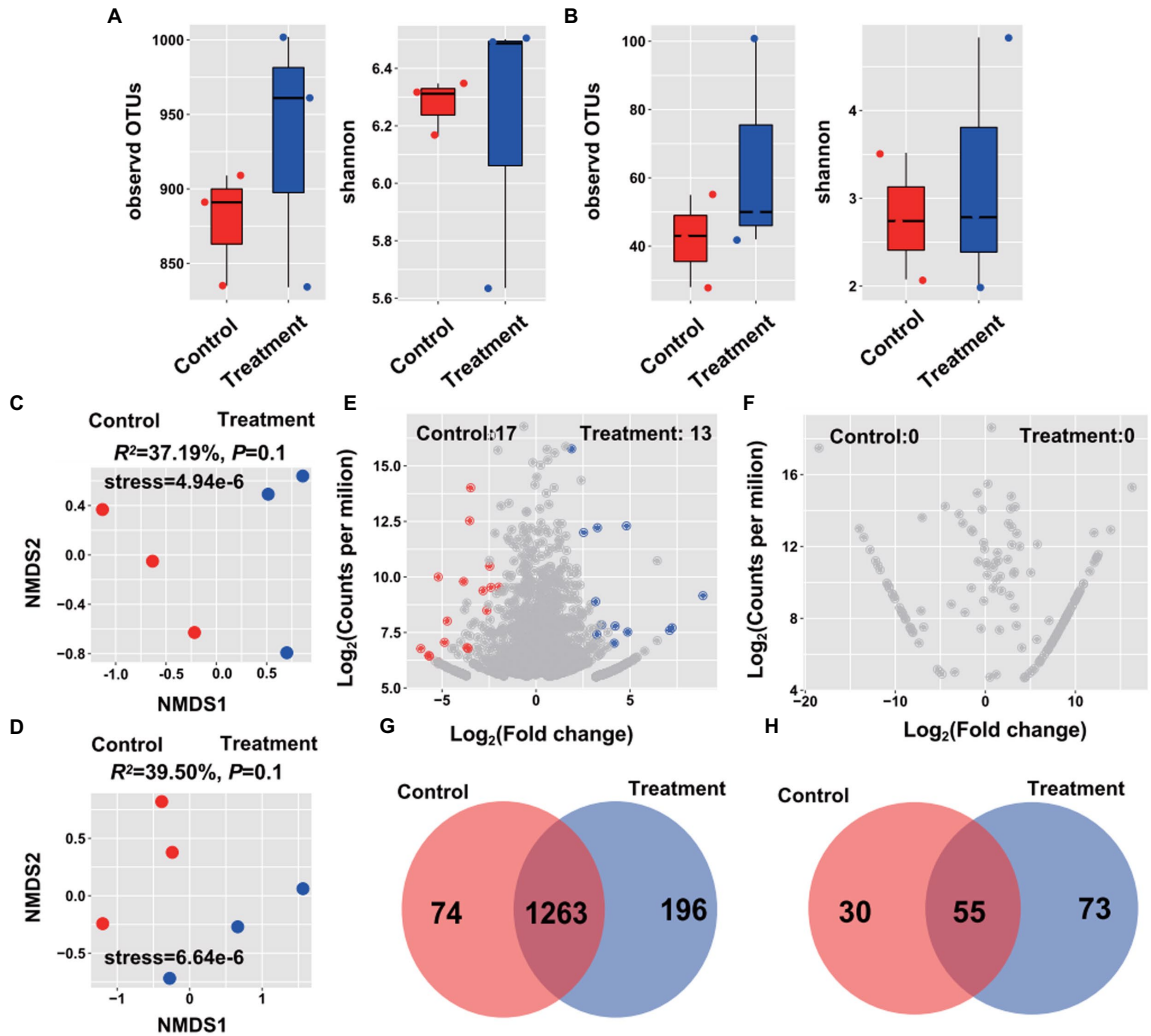
Influence of Ej2 on rice root bacterial and fungal community. The alpha-diversity index was based on the bacterial **(A)** and fungal **(B)** endophytic community in the control and Ej2-inoculation. Non-metric multidimensional scaling (NMDS) ordinations of bacterial **(C)** and fungal **(D)** communities across all root samples. Volcano plot illustrating the enriched OTUs in the bacterial **(E)** and fungal **(F)** microbiome in the control and Ej2-inoculated samples. Venn diagrams showing the shared and specific OTUs of bacterial **(G)** and fungal **(H)** microbiomes in the control and Ej2-inoculated samples.

Alpha-diversity of root endophytes was measured by the Shannon and Observed OTUs indices. The Ej2-inoculated plants exhibited a higher alpha-diversity compared to the control plants, expect for the Shannon index of the bacterial microbiome. However, no differences between control and Ej2-inoculated plants were observed in the bacterial and fungal microbiome alpha-diversity (Figures 3A,B). Furthermore, the NMDS revealed that the Ej2-inoculated samples clustered and separated from the control samples (*R^2^* = 37.19% and *R^2^* = 39.50%, *p* = 0.1, Figures 3C,D). Based on the OTU classification, Gammaproteobacteria (47.12%), Alphaproteobacteria (43.54%), Bacteroidetes (4.85%), Actinobacteria (2.90%), and Firmicutes (1.23) were the dominant phyla in the root bacterial endophytic community of Ej2-inoculated and control samples. In comparison, Sordariomycetes (20.58%) and Dothideomycetes (19.59%) were the dominant classes of the fungal endophytic community (Supplementary Figures S2A,B). The dominant genera of the bacterial microbiome included *Pseudomonas*, *Hydrogenophaga*, *Devosia*, *Mesorhizobium*, and *Shinella*, while *Preussia*, *Fusarium*, *Podospora*, *Olpidium*, and *Schizothecium* were the dominant genera for the fungal community. Compared with the control, strain Ej2 inoculation resulted in a significant enrichment in Alphaproteobacteria, while the control plants were enriched in Gammaproteobacteria and Dothideomycetes (Welch’s test, Supplementary Figures S2C,D).

To examine the effect of Ej2 inoculation on bacterial and fungal communities under salt stress, we identified specific and significantly enriched OTUs in control and Ej2-inoculated plants. The Ej2-inoculated and control plants possessed 13 and 17 enriched bacterial microbiome OTUs, while there was no enriched fungal microbiome OTUs (Figures 3E,F). Interestingly, Ej2-inoculated plants possessed more specific bacterial and fungal microbiome OTUs than the control plants (Figures 3G,H). Furthermore, we analyzed the microbial compositions of enriched and specific OTUs to obtain a detailed overview of the Ej2 inoculation effects. The enriched OTUs after Ej2 inoculation were annotated as *Pseudomonas*, *Flavobacterium*, *Bauldia*, *Mesorhizobium*, *Devosia*, *Raoultella*, *Actinoplanes*, *Yeosuana*, and *Mariniflexile* and the enriched OTUs of the control belonged to *Leucobacter*, *Bacillus*, *Pseudomonas*, *Sphingosinicella*, *Devosia*, *Sphingobium*, *Methyloversatilis*, *Hydrogenophaga*, *Kerstersia*, *Kribbella*, and *Comamonas* at the genus level (Supplementary Table S2). Notably, specific OTUs after Ej2 inoculation were affiliated with *Streptomyces*, *Pseudomonas*, *Flavobacterium*, and *Bacillus* at the genus level. These results suggested that the Ej2 inoculation significantly affects the root endophytic microbiome composition.

### Proteome profile of rice roots inoculated with strain Ej2 in response to salt stress

**Figure 4:**
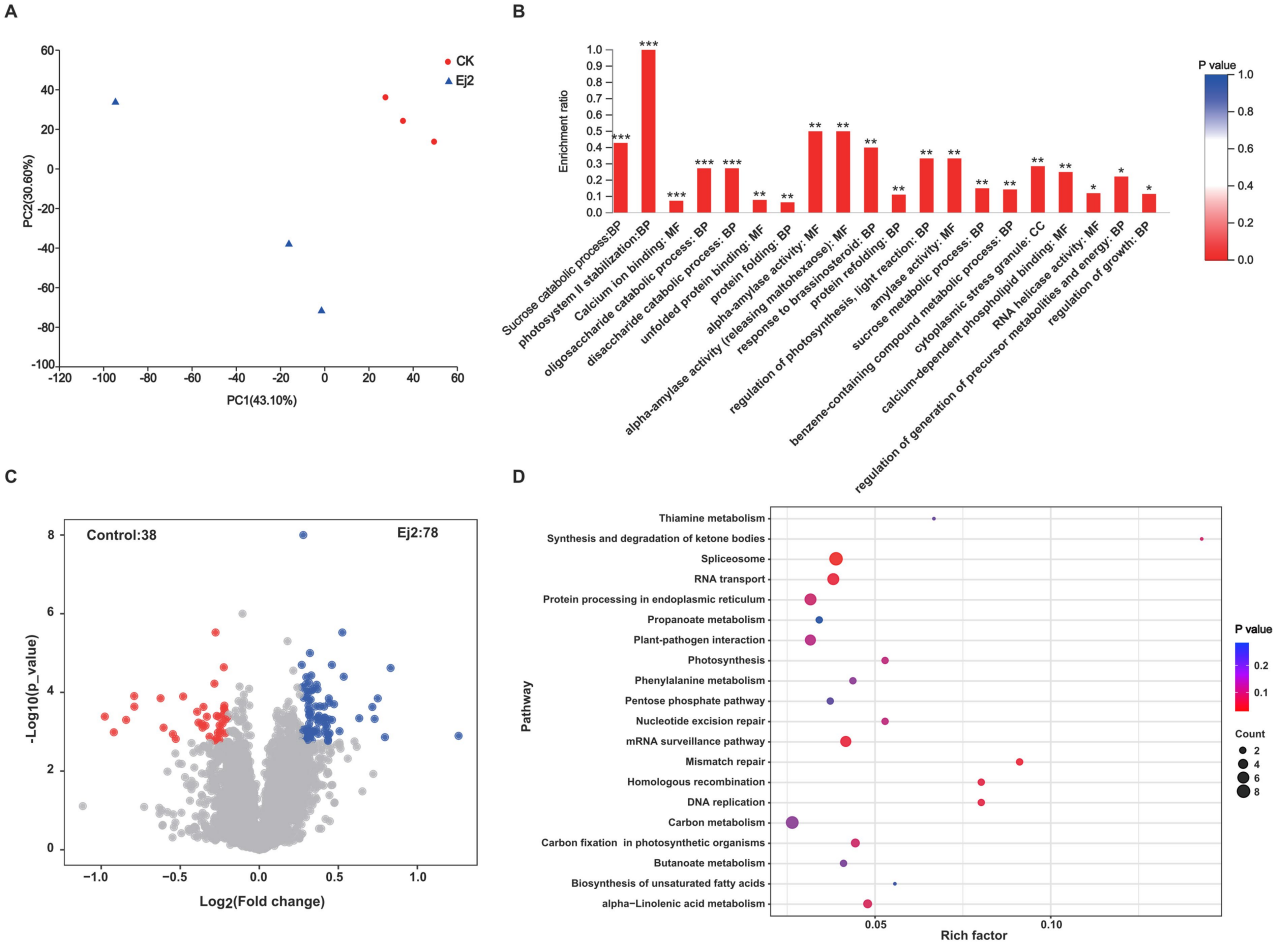
Proteomic profile of rice roots inoculated with strain Ej2 in response to salt stress. **(A)** Principal component analysis of the Ej2-treated and control group under salt stress. **(B)** GO enrichment analysis of all differentially expressed proteins between the control and Ej2 inoculation group. ***p < 0.001, **p < 0.01. **(C)** Identification of differentially expressed proteins between control and Ej2-inoculated rice plants. Red points represent downregulated proteins, blue represent upregulated proteins, and nosing gray represents unchanged proteins. **(D)** KEGG enrichment analysis of all differentially expressed proteins between the control and Ej2 inoculation group. Only the top 20 pathways are displayed.

A proteomic approach was used to identify the proteins related to salt tolerance in rice roots inoculated with Ej2 under salt stress. Firstly, the PCA analysis showed that the two groups of samples could be clearly separated. The two main coordinates explained 73.70% of the variation, with PC1 accounting for 43.10%, and PC2 for 30.60% of the total variation (Figure 4A). Then, we identified the differentially expressed proteins (DEPs) for Ej2-inoculated and control plants. A total of 116 differentially expressed proteins were identified between the control and Ej2-inoculated plants, including 78 upregulated and 38 downregulated proteins. Volcano plots were generated to visualize the distribution of protein expression variation (Figure 4C).

To better understand the biological process altered by Ej2 inoculation under salt stress, the differentially abundant proteins were functionally classified using GO annotation. In the biological process, gene ontology, sucrose catabolic, photosystem II stabilization, oligosaccharide catabolic, disaccharide catabolic, protein binding, response to brassinosteroid, protein refolding, regulation of photosynthesis, light reaction, sucrose metabolic, benzene-containing compound metabolic, regulation of growth, and regulation of generation of precursor metabolites and energy were notably enriched. Only cytoplasmic stress granule was significantly enriched in the cellular component gene ontology. In the molecular functions, gene ontology, calcium ion binding, unfolded protein binding, alpha-amylase activity, calcium-dependent phospholipid binding, and RNA helicase activity were enriched dramatically (Figure 4B). In addition to the functional GO annotation, KEGG pathway analysis was also conducted on the significant DEPs, to provide a comprehensive understanding of the proteomic profile after Ej2 inoculation under salt stress. Most pathways related to metabolism included thiamine, propanoate, phenylalanine, carbon, butanoate, and alpha-Linolenic acid metabolism, plant-pathogen interactions, biosynthesis of unsaturated fatty acids; protein processing in the endoplasmic reticulum, RNA transport, nucleotide excision repair, and homologous recombination. Only the spliceosome pathway was significantly enriched (Figure 4D).

## Discussion

In the present study, the *P. alcaliphila* Ej2 strain was isolated from rice leaves and assayed for its biocontrol and plant-growth-promoting properties under salt stress. Strain Ej2 exhibited significant properties in terms of disease control, rice growth promotion, and regulating the structure of the rice endophytic microbiome and protein expression. These findings provide comprehensive evidence that strain Ej2 contributes to salt stress alleviation in rice plants. Unraveling the mechanisms underlying salt tolerance in plants will improve our capacity to achieve a more efficient way to ameliorate salinity stress.

### Ej2 is a potential biocontrol and plant growth-promoting strain for rice

Using antagonistic microorganisms or microbe-derived bioactive compounds to control plant diseases is a suitable alternative and sustainable method of plant protection (Chakraborty et al., 2021). Microorganisms such as *Bacillus* spp., *Pseudomonas* spp., and *Streptomyces* spp., have been identified as biocontrol agents to control rice blast (Wu et al., 2018; Chaiharn et al., 2020; Zhou et al., 2021a). However, the major challenge when selecting biocontrol agents that appear effective in *in vitro* experiments is that they might not be effective in controlling plant diseases in field conditions (Law et al., 2017). In the present study, strain Ej2 showed a stronger antagonistic effect to rice blast in greenhouse and field experiments. While the efficacy of Ej2 in different experimental site displayed discrepancy, and the prevention effect of Ej2 in Wuzhong was better than in the Lingwu site (Figure 1D). The efficacy of biocontrol agents is affected by the soil’s organic matter, pH, and moisture (Law et al., 2017). Because of the variations of environmental conditions in different locations, the biocontrol agent performance might show variation in the field experiments. Moreover, the formulation and application method can also affect the outcomes of field experiments. A seed coating treatment consisting of *Paenibacillus alvei* K-165 xanthan gum and talc was the most effective in reducing disease symptoms compared to other treatment (Schoina et al., 2011). The promotion effect of biocontrol strain *Bacillus tequilensis* JN-369 was highest when the concentration of JN-369 suspensions reached 10^8^ CFU/ml (Zhou et al., 2021b). The appropriate application methods, therefore, play a vital role in the success of biocontrol agents in the field. As a result, we need to confirm the biocontrol efficacy of Ej2 under different treatments in the future.

### Impact of the biocontrol agent on the endophytic community for salt tolerance improvement

Plant sustain enormous diverse communities of microorganisms, the plant microbiome, important for plant health and robustness and tolerance to biotic and abiotic stresses (Mendes et al., 2013). There is an increasing recognition that plant stress tolerance relates to their associated microbiome. For example, upon *Diaporthe citri* infection, the phyllosphere microbiome shifted and was enriched with beneficial microbes (Li et al., 2022). The salinity-induced changes in rhizosphere microbial communities tend to promote *Hibiscus hamabo* germination and growth (Yuan et al., 2019). Drought triggered a compartment-specific restructuring of rice root microbiota, with endosphere communities displaying a more pronounced response than rhizosphere communities (Santos-Medellin et al., 2017). Studies focusing on synthetic microbial communities have emerged, driven by the great application potential to provide sustainable agriculture solutions. For example, five bacterial strains isolated from the root of the desert plant could protect, as synthetic bacterial communities, tomato plants growing in high salinity (Vorholt et al., 2017; Schmitz et al., 2022). Moreover, organic fertilizers supplemented with the biocontrol strain *B. amyloliquefaciens* W19 improved resistance against plant pathogens due to their impacts on the resident soil microbiome structure and function, specifically increasing *Pseudomonas* species. Furthermore, *B. velezensis* stimulated the resident rhizosphere bacterium *P. stutzeri* through metabolic interactions, and the two-species consortium helped plants alleviate salt stress (Tao et al., 2020; Sun et al., 2022). Microbial products could suppress potato scab disease, increase the yield, and increase the relative abundance of taxa representing beneficial bacteria such as *Agrobacterium*, *Bacillus*, and *Pseudomonas*. Furthermore, the microbial product amount applied significantly influenced the diversity and bacterial community (Wang Z. S. et al., 2019). Finally, the bacterial and fungal communities were significantly altered after applying bio-organic fertilizers in cotton *Verticillium* wilt and decline disease of bayberry (Tian et al., 2016; Ren et al., 2021).

Only selected soil microbiome members may move from soils to the plant and form complex co-associations with plants (Xiong et al., 2021). A previous report found that Proteobacteria, Actinobacteria, Bacteroidetes and Gemmatimonadetes was dominant in saline soil (Canfora et al., 2014; Kadam and Chuan, 2016). In our study, we identified Gammaproteobacteria, Alphaproteobacteria, Bacteroidetes, Actinobacteria, and Firmicutes as the dominant classes (Supplementary Figure S2). They showed remarkable convergence at the phylum level and lower taxonomic ranks with differences among soil and plant. Furthermore, the relative abundance of Proteobacteria and Bacteroidetes was positively, and Acidobacteria was negatively correlated with salinity (Canfora et al., 2014). The OTUs annotated as Gammaproteobacteria and Alphaproteobacteria showed a high-salinity niche preference (Zhang et al., 2019). Our results revealed that the Ej2 inoculation significantly enriched Alphaproteobacteria, while the control plants were enriched in Gammaproteobacteria (Supplementary Figure S2). Thus, the differences are potentially caused by plant compartment niches. Based on the enriched and specific OTUs results, we found that the enriched OTUs belonging to *Pseudomonas* and *Flavobacterium* at the genus level (Figure 3). The *Flavobacterium* probably plays an important role in salt tolerance for plants. But this needs to be confirmed in the further study. Network analysis and synthetic community experiments have identified some key taxa that play a central role in the microbiome’s structure and function, such as *Bacillus*, *Streptomyces*, *Rhizobium*, and *Flavobacterium* (Xiong et al., 2021). In addition, *B. velezensis* JC-K3 significantly altered the diversity and abundance of endophytic microorganisms in wheat, increasing the diversity of bacterial communities in the leaves (Ji et al., 2021). Similarly, we found that the inoculation increased the diversity in our study (Figure 1).

### The biocontrol strain help plants to alleviate salt stress

Plants are multi-cellular organisms that cope with various environmental stresses by producing and accumulating diverse functional compounds (proline, glycine betaine, phenolic, flavonoids and glutathione), and enzymatic antioxidants (peroxidase and catalase), which mitigate the oxidative damage caused by high salinity. Furthermore, many studies have documented that microbes have beneficial activities such as reducing pathogenesis, stimulating plant growth, and alleviating abiotic stresses. Significant differences were observed in inoculated plants’ antioxidant enzymes (ascorbate peroxidase, catalase and peroxidase), proline content, and total antioxidative capacity (Fan et al., 2020). For example, the expression of salt stress-responsive genes related to proline biosynthesis was upregulated in *Arabidopsis* after short-term treatment with *Enterobacter* sp. (Kim et al., 2014). In addition, several salinity-responsive marker genes, such as *AtRSA1*, involved in detoxifying reactive oxygen species, and *AtWRKY8* and *AtVQ9*, involved in maintaining ion homeostasis, were increased (Sukweenadhi et al., 2015). *Alcaligenes faecalis* emits hexanedioic acid and butanoic and induces plant salt tolerance through reprogramming the auxin and gibberellin pathways (Bhattacharyya et al., 2015). In addition, proteomic analysis revealed that the expression levels of diverse proteins involved in photosynthesis, antioxidative processes, transportation across membranes, and pathogenesis-related responses were altered in the presence of microbes (Cheng et al., 2012). *P. fluorescens* helped canola plants endure salinity through the enrichment of proteins related to energy metabolism and cell division, especially related to amino acid metabolism and the tricarboxylic acid cycle (Banaei-Asl et al., 2015). In our study, we only found that the spliceosome pathway was significantly enriched. However, other biological process gene ontologies such as sucrose catabolic, regulation of photosynthesis, sucrose metabolic, benzene-containing compound metabolic, regulation of growth, and regulation of generation of precursor metabolites and energy were notably enriched (Figures 4B,D). Elucidating the mechanisms of Ej2 underlying salt tolerance in plants will help us to develop more efficient products for saline soil amelioration.

## Data availability statement

The amplicon data presented in the study are deposited in the NCBI Sequence Read Archive (SRA) repository, accession number PRJNA904920 and the mass spectrometry proteomics data presented in this study are deposited in the ProteomeXchange Consortium (http://proteomecentral.proteomexchange.org), accession number PXD039112.

## Author contributions

YS and QZ designed the experiment. QZ, XM, and LZ analyzed the data and wrote the manuscript. YS, ZH, and HY performed the filed experiments and collected samples. QZ, XM, ZH, LZ, HY, and YS approved the manuscript. All authors contributed to the article and approved the submitted version.

## Funding

This work was supported by the Natural Science Foundation Project of Ningxia (2020AAC03317), the Key Research and Development Program of Ningxia (2022BBF02031), the China Postdoctoral Science Foundation (2019M660508), and the Young Top-notch Talent Training Project of Ningxia.

## Conflict of interest

The authors declare that the research was conducted in the absence of any commercial or financial relationships that could be construed as a potential conflict of interest.

## Publisher’s note

All claims expressed in this article are solely those of the authors and do not necessarily represent those of their affiliated organizations, or those of the publisher, the editors and the reviewers. Any product that may be evaluated in this article, or claim that may be made by its manufacturer, is not guaranteed or endorsed by the publisher.

## Footnotes

^1^ https://cloud.oebiotch.cn

^2^ http://asia.ensembl.org/Mus_musculus/Info/Index

^3^ http://www.blast2go.com/b2ghome

^4^ http://geneontology.org/

^5^ http://www.genome.jp/kegg/

^6^ www.majorbio.com

## Supplementary material

Supplementary Figure S1 Plant growth-promoting activities of Ej2 at different concentrations of NaCl. **(A)** Emergence rate, **(B)** relative water content of the leaf, **(C)** fresh root weight, and **(D)** dry rot weight. The significance test was performed based on a t-test using Prism 9. *: *p* < 0.05, **: *p* < 0.01.

Supplementary Figure S2 Relative abundance of the dominant bacterial **(A)** and fungal **(B)** taxa in control and treatment samples. Differential abundance analysis of bacterial **(C)** and fungal **(D)** OTUs in control and Ej2-inoculated samples. Welch’s test was performed to calculate the significance of differences at the phylum and class levels.

Supplementary Table S1 In vitro antifungal activity of Ej2 against various phytopathogens on PDA plates.

Supplementary Table S2 Comparison of enriched OTUs between the control and Ej2 inoculation.
